# An attenuated mutant of the Rv1747 ATP-binding cassette transporter of *Mycobacterium tuberculosis* and a mutant of its cognate kinase, PknF, show increased expression of the efflux pump-related *iniBAC* operon

**DOI:** 10.1111/1574-6968.12230

**Published:** 2013-08-23

**Authors:** Vicky L Spivey, Rachael H Whalan, Elizabeth M A Hirst, Stephen J Smerdon, Roger S Buxton

**Affiliations:** 1 Division of Mycobacterial Research, MRC National Institute for Medical ResearchLondon, UK; 2 Division of Developmental Neurobiology, MRC National Institute for Medical ResearchLondon, UK; 3 Division of Molecular Structure, MRC National Institute for Medical ResearchLondon, UK; Horizon Discovery Ltd.Building 7100, IQ Cambridge, Waterbeach, Cambridge, CB25 9TL, UK

**Keywords:** mycobacteria, virulence, serine/threonine protein kinase, transcriptomics, DNA microarray, isoniazid

## Abstract

The ATP-binding cassette transporter Rv1747 is required for the growth of *Mycobacterium tuberculosis* in mice and in macrophages. Its structure suggests it is an exporter. *Rv1747* forms a two-gene operon with *pknF* coding for the serine/threonine protein kinase PknF, which positively modulates the function of the transporter. We show that deletion of *Rv1747* or *pknF* results in a number of transcriptional changes which could be complemented by the wild type allele, most significantly up-regulation of the *iniBAC* genes. This operon is inducible by isoniazid and ethambutol and by a broad range of inhibitors of cell wall biosynthesis and is required for efflux pump functioning. However, neither the *Rv1747* or *pknF* mutant showed increased susceptibility to a range of drugs and cell wall stress reagents including isoniazid and ethambutol, cell wall structure and cell division appear normal by electron microscopy, and no differences in lipoarabinomannan were found. Transcription from the *pknF* promoter was not induced by a range of stress reagents. We conclude that the loss of Rv1747 affects cell wall biosynthesis leading to the production of intermediates that cause induction of *iniBAC* transcription and implicates it in exporting a component of the cell wall, which is necessary for virulence.

## Introduction

The increase in multidrug and extensively drug-resistant *Mycobacterium tuberculosis* strains has made the search for new TB drugs ever more important. Deciphering the function of important *M. tuberculosis* proteins is a key strategy to identify potential new drug targets. Rv1747 is an ATP-binding cassette (ABC) transporter that is required for the growth of *M. tuberculosis* in macrophages, dendritic cells and mice (Curry *et al*., 2005). A total of 37 ABC transporters have been identified in *M. tuberculosis*; 16 have been categorised as importers and 21 as exporters (Braibant *et al*., 2000).

ABC transporters are integral membrane proteins comprising two transmembrane domains and two cytoplasmic nucleotide-binding domains; they bind and hydrolyse ATP providing energy for uptake or export of substrates across cell membranes. Functions include the uptake of nutrients into the cells and the export of virulence factors and toxins (Holland *et al*., 2003). Bacterial ABC importers are typically formed from four polypeptide chains that are often separately encoded (Saurin *et al*., 1999) and require an external binding protein, which functions to deliver the substrate to the transporter (Dawson *et al*., 2007). In contrast, bacterial exporters are produced as one polypeptide where a single gene usually encodes both the transmembrane domain and a nucleotide-binding domain (Saurin *et al*., 1999). The presence of fused nucleotide-binding and transmembrane domains is a strong indicator of an ABC exporter (Dawson *et al*., 2007; Davidson *et al*., 2008). Based on its amino acid sequence, Rv1747 belongs to the G subfamily of ABC transporters; this family consists of half-transporters, which oligomerise to form the functional transporter. Furthermore, this protein encodes both the transmembrane and nucleotide-binding domains in one polypeptide and is thus probably an exporter. *Rv1747* forms a two-gene operon with its upstream adjacent gene, the serine–threonine protein kinase (STPK) *pknF* (Spivey *et al*., 2011).

Interestingly, Rv1747 also contains two forkhead-associated (FHA) domains; these are modular phosphopeptide recognition motifs, and their presence is taken to indicate that the protein is likely to interact with a phosphorylated protein partner (Durocher & Jackson, 2002; Spivey *et al*., 2011). Rv1747 exhibits ATPase activity and is a substrate for PknF *in vitro*; furthermore, both FHA domains of Rv1747 are required for this interaction in a yeast two-hybrid assay (Molle *et al*., 2004; Curry *et al*., 2005). More recently, we have identified specific threonine residues on Rv1747 that are phosphorylated, at least *in vitro*, by PknF and which have *in vivo* modulatory effects on the function of the Rv1747 ABC transporter (Spivey *et al*., 2011).

Bacterial ABC exporters transport many different substances including cell surface components such as lipopolysaccharides, lipids, proteins involved in pathogenesis, peptides, drugs and siderophores (Dassa, 2003). In *M. tuberculosis,* one ABC transporter, DrrABC and an RND family transmembrane protein, MmpL7, are required for the translocation to the cell surface of phthiocerol dimycocerosates (PDIMs), complex lipids required for virulence (Cox *et al*., 1999; Camacho *et al*., 2001); interestingly, MmpL7 is a potential substrate of another STPK, PknD (Pérez *et al*., 2006). Rv1747 could export any one of these molecules, which would make the function of the transporter important for growth *in vivo*. Rv1747 falls into a subclass of *M. tuberculosis* ABC transporters, which have an unknown function (Braibant *et al*., 2000). Similarity was found to the White protein from *Drosophila melanogaster*, a permease necessary for the transport of pigment precursors responsible for eye colour, and to NodI from *Rhizobium* strains, a protein implicated in the nodulation process by export of a polysaccharide (Braibant *et al*., 2000).

We have investigated the phenotypic consequences of the loss of the Rv1747 and PknF proteins in deletion mutants. Significantly, using transcriptional profiling, we demonstrate that the expression of the *iniBAC* operon is up-regulated in both mutants.

## Materials and methods

### Bacterial strains and growth conditions

*Mycobacterium tuberculosis* H37Rv and *Escherichia coli* K-12 strains are described in Table 1. Growth conditions have been described previously (Spivey *et al*., 2011).

**Table 1:** Bacterial strains and plasmids

Strains or plasmids	Genotype or description	Source or reference
*E. coli* strains		
* E. coli* TOP10	F^−^ *mcr*A Δ(*mrr*-*hsd*RMS-*mcr*BC) φ80*lacZΔM15* Δ*lacX74 deoR recA1 araD139* Δ(*ara*-*leu*)7697 *galU galK rpsL endA1 nupG*; used for general cloning	Invitrogen
*M. tb*. strains		
* *H37Rv	*M. tuberculosis* WT strain	Oatway & Steenken (1936)
* *Δ*pknF*	H37Rv with deletion of *pknF* constructed by homologous recombination with targeting construct pRW51	Spivey *et al*. (2011)
* pknF* complement	Δ*pknF* containing complementing plasmid pRW95	Spivey *et al*. (2011)
* *Δ*Rv1747*	H37Rv with deletion of *Rv1747* constructed by homologous recombination with targeting construct pRW69	Curry *et al*. (2005)
* Rv1747* complement	Δ*Rv1747* containing complementing plasmid pRW76	Curry *et al*. (2005)
*M. tuberculosis* shuttle plasmids		
* *p2Nil	Suicide gene delivery vector, *oriE,* Kan^R^	Hinds *et al*. (1999)
* *pKP186	Integrase negative derivative of the integrating vector pMV306, Kan^R^	Papavinasasundaram *et al*. (2001)
* *pBS-Int	Suicide vector containing integrase, Amp^R^	Springer *et al*. (2001)
* *pEJ414	pMV306 derivative containing a promoterless *E. coli lacZ* reporter gene, Kan^R^	Papavinasasundaram *et al*. (2001)
* *pRW69	p2Nil containing a 2-kb region of H37Rv DNA flanking each side of the *Rv1747* gene, Hyg^R^	Curry *et al*. (2005)
* *pRW76	*Rv1747* complementing plasmid. pKP186 derivative containing 609 bp *Rv1745c*,*pknF* and *Rv1747*, Kan^R^ Hyg^R^	Curry *et al*. (2005)
* *pRW51	p2Nil containing a 1.5-kb region of H37Rv DNA flanking each side of the *pknF* gene	Spivey *et al*. (2011)
* *pRW95	*pknF* complementing plasmid. pKP186 derivative containing 609 bp *Rv1745c*,*pknF* and 20 bp of *Rv1747*, Kan^R^	Spivey *et al*. (2011)
* *pVS_01	pEJ414 containing *pknF* promoter region, Kan^R^	This work

### Generation of the *pknF* and *Rv1747* deletion and complementing strains

The construction of the *pknF* (*Rv1746*) null strain (*ΔpknF*) was described previously (Spivey *et al*., 2011); this has an in-frame unmarked deletion to avoid downstream polar effects on the *Rv1747* gene. For complementation of the *pknF* deletion, the genes *pknF*, 609 bp of *Rv1745c* and 20 bp of *Rv1747* were amplified by PCR (Spivey *et al*., 2011) and the product cloned into the vector pKP186 (Rickman *et al*., 2005), a pMV306 (Kong & Kunimoto, 1995) derivative lacking the integrase gene and electroporated into the *ΔpknF* mutant along with the mycobacterial suicide vector, pPS-Int containing the integrase gene (Springer *et al*., 2001; Curry *et al*., 2005). Construction of the hygromycin-marked *Rv1747* deletion mutant was described previously (Curry *et al*., 2005). For complementation of the *Rv1747* deletion, the genes *Rv1747*,*Rv1746 (pknF)*, and 609 bp of *Rv1745c* were amplified by PCR (Curry *et al*., 2005), cloned into the vector pKP186 (Rickman *et al*., 2005) and transformed into the *ΔRv1747* mutant.

### cDNA labelling and microarray analysis

RNA isolation from *M. tuberculosis* liquid cultures was described previously (Spivey *et al*., 2011). Whole genome DNA microarrays of *M. tuberculosis* (version 2) were provided by the BμG@S group (St. George's, University of London). cDNA labelling and RNA–DNA microarray hybridisations were described previously (Rickman *et al*., 2005). Microarray slides were scanned as previously (Hunt *et al*., 2008), grids were fitted using bluefuse software and analysed using genespring, version 10 (Agilent). Three biological replicates were performed for each condition, carried out in duplicate for dye swaps. The genes described only include those whose differential gene regulation was restored to wild type (WT) in complemented mutants. The array design is available in BμG@Sbase (Accession No. A-BUGS-23; http://bugs.sgul.ac.uk/A-BUGS-23) and ArrayExpress (Accession No. A-BUGS-23). Fully annotated microarray data have been deposited in BμG@Sbase (accession number E-BUGS-149; http://bugs.sgul.ac.uk/E-BUGS-149) and ArrayExpress (accession number E-BUGS-149).

### Quantitative real-time PCR (qRT-PCR)

cDNA was generated from 1 μg RNA using the Quantitect reverse transcription kit (Qiagen). Primers (Table 2) were designed using primer express 3.0 (Applied Biosystems). Real-time quantitative PCR analysis on this cDNA was performed using the ABI Prism 7500 using the Fast SYBR green master mix (Applied Biosystems). Data were normalised to *sigA* expression.

**Table 2:** Primers used for qRT-PCR

Primer name	Sequence (5′-3′)
*pknF F*	CACGAACGTCGGCTGTTG
*pknF R*	GACGATCAGGTGAATCAGGATTG
*Rv1747 F*	TACGGTCGACCTGATCAAATTG
*Rv1747 R*	GCGCTGGCGGTGTGA
*iniA F*	TCATCGCAGTCTCATCACTGTTG
*iniA R*	TTGGACTCTTCGTTGAGCTCTTT
*iniB F*	TTATCGATTACATCCTGAGCCTGTT
*iniB R*	CGGAGCGGCAACGAA
*iniC F*	ACTCCGAATGCTAAGCCTTTTG
*iniC R*	CAGCGACGCGATTTCGT
*ethA F*	GCAAGCCCATCCTCGAGTAC
*ethA R*	CGGATATGCCTGTCGATTCC
*pknD F*	CAACGGACAGTTCTTTGTCGAA
*pknD R*	TGTTTCAATAGGGCGCGTAAA
*sigA F*	TCGGTTCGCGCCTACCT
*sigA R*	GGCTAGCTCGACCTCTTCCT

Other methods are described in Supporting information, Appendix S1.

## Results

### Transcriptional microarray analysis of the *ΔpknF* and *ΔRv1747* mutants

Transcriptional microarray analysis was performed to compare gene expression in WT H37Rv vs. *ΔRv1747*, WT vs. *ΔpknF*, WT vs. the *Rv1747* complemented mutant and WT vs. the *pknF*-complemented mutant. The top 10 most highly regulated genes whose differential expression was restored by complementation are shown in Table 3. As expected, expression of *Rv1747* was 29-fold down-regulated in the mutant (compared with WT). In the *Rv1747* complemented strain, expression of *Rv1747* was 1.3-fold up-regulated compared with WT, confirming restoration of gene expression.

**Table 3:** Microarray data for the 10 *Mycobacterium tuberculosis* genes most highly up- and down-regulated upon *Rv1747* deletion

Rv number	Gene name	Gene product	Fold regulated	*P*-value
*Rv0005*	*gyrB*	DNA gyrase subunit B	2.0 up	0.001
*Rv0046c*	*ino1*	Myo-inositol-1-phosphate synthase Ino1	2.0 up	3.00E−04
*Rv0047c*	*Rv0047c*	Conserved hypothetical protein	2.2 up	4.10E−05
*Rv0341*	*iniB*	Isoniazid-inducible gene protein IniB	3.8 up	2.40E−04
*Rv0342*	*iniA*	Isoniazid-inducible gene protein IniA	3.2 up	8.10E−05
*Rv0822c*	*Rv0822c*	Conserved hypothetical protein	2.0 up	4.00E−05
*Rv1040c*	*PE8*	PE family protein	2.1 down	0.013
*Rv1380*	*pyrB*	Probable aspartate carbamoyltransferase PyrB	2.2 down	0.001
*Rv1382*	*Rv1382*	Probable export or membrane protein	2.2 down	6.20E−04
*Rv1747*	*Rv1747*	Probable ABC transporter	29.2 down	8.90E−06
*Rv1999c*	*Rv1999c*	Probable conserved integral membrane protein	2.2 down	0.004
*Rv2007c*	*fdxA*	Probable ferredoxin FdxA	2.1 up	0.005
*Rv2265*	*Rv2265*	Possible conserved integral membrane protein	2.1 down	0.005
*Rv2396*	*PE_PGRS41*	PE-PGRS family protein	2.0 up	7.00E−04
*Rv2415c*	*Rv2415c*	Conserved hypothetical protein	2.3 down	7.90E−04
*Rv2528c*	*mrr*	Probable restriction system protein Mrr	2.1 down	1.90E−04
*Rv2577*	*Rv2577*	Conserved hypothetical protein	2.1 down	0.015
*Rv2814c*	*Rv2814c*	Probable transposase	2.2 down	0.011
*Rv3140*	*fadE23*	Probable acyl-CoA dehydrogenase FadE23	2.1 up	0.001
*Rv3854c*	*ethA*	Monooxygenase EthA	2.0 up	0.003

The gene most up-regulated (3.8-fold) in the Δ*Rv1747* strain was the isoniazid-inducible gene *iniB*, whilst the second most up-regulated was *iniA* (3.2-fold). *iniB* forms an operon with *iniA* and *iniC,* which was up-regulated 1.6-fold. Other genes in the top 10 list of up-regulated genes included the probable acyl-CoA dehydrogenase *fadE23* (2.1-fold) and a probable ferredoxin, *fdxA* (2.1-fold). Genes with a 2.0-fold up-regulation in the mutant were *gyrB* (DNA gyrase subunit B), PE_PGRS41 (Rv2396; a PE_PGRS family protein), *ethA* (whose gene product activates the prodrug ethionamide), *ino1* [involved in the phosphatidyl-myo-inositol (PI) biosynthetic pathway] and two conserved hypothetical proteins, Rv0047c and Rv0822c. Of the STPKs, *pknF* (1.9-fold) and *pknD* (1.8-fold) were up-regulated. *pknF* expression was 2.0-fold up-regulated in the *Rv1747* complement strain, probably because the complementing plasmid contains an intact copy of *pknF*.

Comparison of the WT and *ΔpknF* strains yielded only 12 genes differentially regulated at least twofold, all down-regulated in the mutant. This number increased to 72 with a 1.5-fold cut-off, of which only 12 were up-regulated. The microarray data for the 10 most up- and down-regulated genes in the *pknF* deletion strain are presented (Table 4). The *pknF* gene itself did not appear in the microarray results list because it did not pass the filtering stages within the analysis.

**Table 4:** Microarray data for the 10 *Mycobacterium tuberculosis* genes most highly up- and down-regulated upon *pknF* deletion

Rv number	Gene name	Gene product	Fold regulated	*P*-value
*Rv0175*	*Rv0175*	Probable conserved Mce-associated membrane protein	1.5 up	0.002
*Rv0341*	*iniB*	Isoniazid-inducible gene protein IniB	1.8 up	0.001
*Rv0796*	*Rv0796*	Putative transposase for insertion sequence element IS6110	2.0 down	0.035
*Rv1370c*	*Rv1370c*	Putative transposase for insertion sequence element IS6110	2.0 down	0.042
*Rv1371*	*Rv1371*	Probable conserved membrane protein	2.4 down	0.042
*Rv1372*	*Rv1372*	Conserved hypothetical protein	2.2 down	0.037
*Rv1738*	*Rv1738*	Conserved hypothetical protein	1.6 up	0.005
*Rv2007c*	*fdxA*	Probable ferredoxin fdxA	1.8 up	0.023
*Rv2106*	*Rv2106*	Probable transposase	2.0 down	0.029
*Rv2167c*	*Rv2167c*	Probable transposase	2.0 down	0.015
*Rv2480c*	*Rv2480c*	Possible transposase for insertion sequence element IS6110	2.4 down	0.003
*Rv2515c*	*Rv2515c*	Conserved hypothetical protein	2.0 down	0.034
*Rv2815c*	*Rv2815c*	Probable transposase	2.0 down	0.044
*Rv3640c*	*Rv3640c*	Probable transposase	2.0 down	0.036
*Rv3727*	*Rv3727*	Possible oxidoreductase	1.5 up	0.008
*Rv3728*	*Rv3728*	Probable conserved two domain membrane protein	1.5 up	0.006
*Rv3842c*	*glpQ1*	Probable glycerophosphoryl diester phosphodiesterase GlpQ1	1.8 up	0.006
*Rv3850*	*Rv3850*	Conserved hypothetical protein	1.7 up	0.002
*Rv3854c*	*ethA*	Monooxygenase EthA	1.7 up	0.006
*Rv3864*	*espE*	Esx-1 secretion-associated protein EspE	1.6 up	0.003

Interestingly, the gene most up-regulated in the *ΔpknF* strain was also *iniB* (1.8-fold). *iniA* was also up-regulated 1.5-fold in the *pknF* deletion strain. Other genes present in the *ΔpknF* list that were also up-regulated in the *Rv1747* null strain were *fdxA* (1.8-fold) and *ethA* (1.7-fold). Other genes up-regulated in the *ΔpknF* mutant were *glpQ1*, a probable glycerophosphoryl diester phosphodiesterase (1.8-fold), the conserved hypothetical proteins *Rv3850* (1.7-fold) and *Rv1738* (*espE*), an Esx1 secretion-associated protein (1.6-fold), *Rv3727* (probably involved in cellular metabolism), *Rv3728* (probably involved in an efflux system) and *Rv0175* (a probable conserved Mce-associated membrane protein; all 1.5-fold). The top 10 list of genes down-regulated in the *ΔpknF* strain comprised seven possible transposases; *Rv2480c* (2.4-fold), and *Rv1380*,*Rv2106*,*Rv3640c*,*Rc2815c*,*Rv2167c*,*Rv0796* (all 2.0-fold). The remaining three down-regulated genes were *Rv1371* (2.4-fold; conserved membrane protein) and *Rv1372* (2.2-fold) and *Rv2515c* (2.0-fold), both annotated as conserved hypothetical proteins.

**Figure 1:**
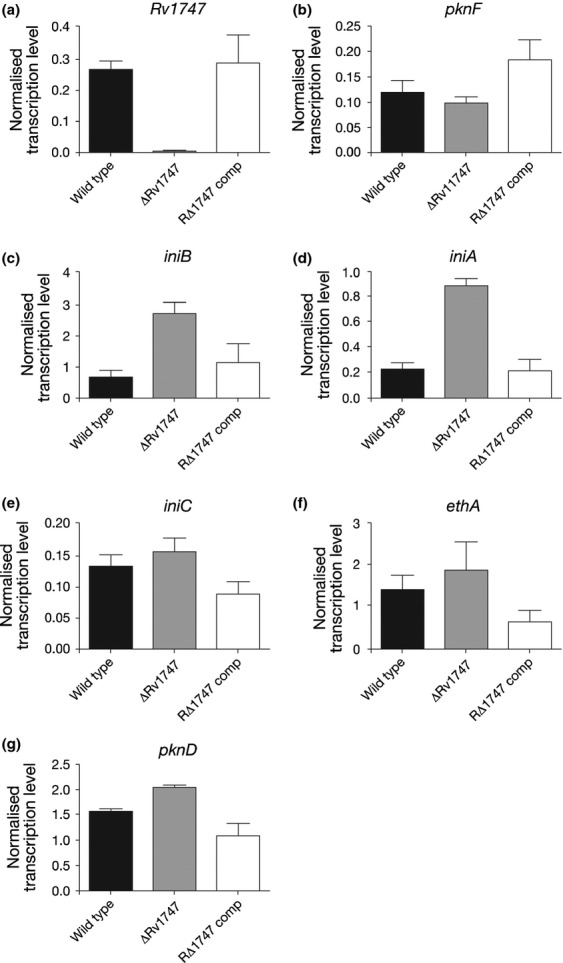
qRT-PCR to confirm the effect of *Rv1747* deletion and complementation on the relative transcription levels of selected genes. Each panel shows the normalised transcription level of each gene investigated in WT,*ΔRv1747* and complement strains. Data plotted are the mean of three biological replicates, and the error bars show the standard deviations. Data were normalised to *sigA* expression.

Confirmation of the microarray results for selected genes was obtained using qRT-PCR. The results (Fig. 1) confirmed that expression of *Rv1747* in the deletion mutant was virtually undetectable and transcription was restored to WT levels in the complementing strain. Expression of *pknF* did not increase in the *ΔRv1747* mutant, but did increase in the complementing strain, as mentioned above. Unlike the microarray data, the level of *pknF* transcript did not increase in the *ΔRv1747* strain when determined by qRT-PCR. The transcriptional profiles of *iniA*,*iniB*,*iniC*,*ethA* and *pknD* all follow the same expression pattern as shown by the microarray results: transcript levels were increased in the *Rv1747* mutant strain and were complemented when the *Rv1747* gene was replaced. This was particularly striking for *iniB* and *iniA* where there was approximately three times as much transcript present in the mutant strain compared with the WT.

**Figure 2:**
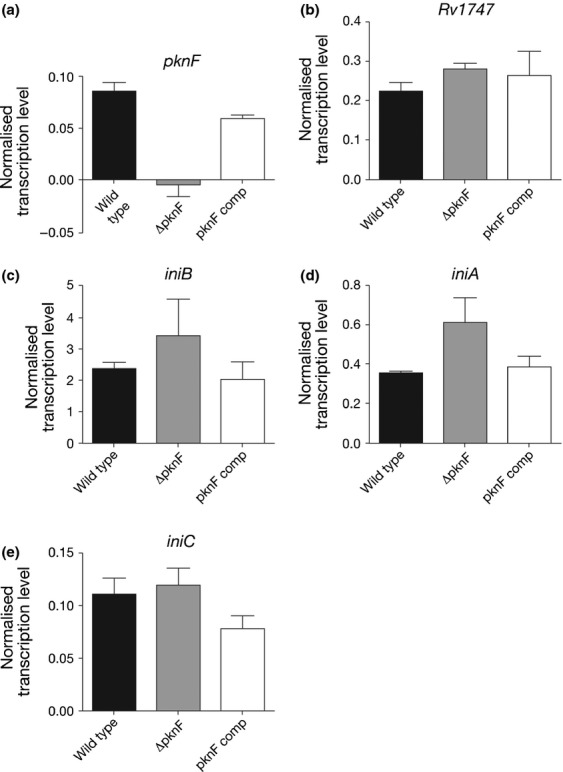
qRT-PCR to confirm the effect of *pknF* deletion and complementation on the relative transcription levels of selected genes. Each panel shows the normalised transcription level of each gene investigated in WT,*ΔpknF* and complement strains. Data plotted are the mean of three biological replicates, and the error bars show the standard deviations. Data were normalised to *sigA* expression.

The results of qRT-PCR for the WT, *ΔpknF* and the *pknF*-complemented strain (Fig. 2) confirmed that expression of *pknF* in the *ΔpknF* mutant was undetectable and transcription was restored to almost WT level in the complementing strain. Expression of *Rv1747* was the same in all three strains; thus, the transcriptional changes seen in the *ΔpknF* mutant are not due to changes in *Rv1747* expression. The transcriptional profiles of *iniB*,*iniA* and *iniC* (Fig. 2c–e) follow the same pattern as shown by the microarray data. The *iniC* gene did not appear in the gene lists generated by microarrays in the *pknF* mutant or complement strain, but Fig. 2e clearly shows that the level of *iniC* transcript did not change in the *ΔpknF* mutant strain but was slightly decreased in the *pknF*-complemented strain.

### The cell wall of the *ΔpknF* and *ΔRv1747* mutants appeared normal by drug sensitivity assays, electron microscopy and lipoarabinomannan content

Many of the genes which were differentially regulated between WT and *ΔpknF* and *ΔRv1747* mutants are involved in cell wall processes. We therefore tested whether the mutants were any more susceptible to cell wall and other stresses, viz. isoniazid, ethambutol, streptomycin, ciprofloxacin, ofloxacin, hydrogen peroxide, S-nitrosoglutathione, ethidium bromide, mitomycin C and sodium dodecyl sulphate, using a microplate Alamar Blue assay. However, no differences were observed in MICs between the mutants and the WT (Fig. S1).

Moreover, the transcription of the *pknF-Rv1747* operon was not altered by isoniazid or ethambutanol (or indeed by a number of other stresses: gentamicin, streptomycin, hydrogen peroxide, t-butyl hydrogen peroxide, diamide, mitomycin C, S-nitrosoglutathione, ofloxacin, plumbagin, sodium nitroprusside, pH 7.2, pH 5.5 or in stationary phase; Fig. S2).

Transmission electron microscopy was performed to examine whether there were any differences in the cell wall structure and composition between the WT, *ΔpknF*,*ΔRv1747* and the respective complementing strains. No discernible differences in cell wall structure were evident between the strains (Fig. S3).

The possibility that Rv1747-transported lipoarabinomannan was tested by analysing this molecule using two anti-lipoarabinomannan antibodies, one primarily recognising not only PIM6 but also ManLAM capped with three mannosyl residues (Mab F183-24) and one recognising the Ara6 structure in lipoarabinomannan (Mab F30-5). There were however no differences in the levels of ManLAM between cell suspensions of the WT, *ΔRv1747* and *Rv1747* complementing strains (Fig. S4).

## Discussion

The substrate of the Rv1747 transporter is presently unknown. Moreover, other than the attenuation of growth of a *ΔRv1747* mutant in mice and macrophages, there are presently no further mutant phenotypes known, growth being normal *in vitro* (Curry *et al*., 2005). We have used transcriptional microarray analysis to see whether this would throw light on the nature of Rv1747 transport function. This has demonstrated significant changes in transcriptional profiles between the *ΔRv1747* and *ΔpknF* mutants and the WT. Interestingly, the genes most up-regulated in both of the mutant strains were in the *iniBAC* operon, identified as isoniazid-inducible (Alland *et al*., 1998). The *iniA* gene was also induced by ethambutol, another *M. tuberculosis* therapeutic agent that also targets the cell wall but with a different mechanism of action. Using *M. bovis* BCG, the promoter of the *iniBAC* operon was shown to be specifically induced by a broad range of inhibitors to cell wall biosynthesis including antibiotics that inhibited the synthesis of peptidoglycan, arabinogalactans, mycolic acids and fatty acids (Alland *et al*., 2000). *iniA* is also essential for the activity of an efflux pump, which confers resistance to isoniazid and ethambutol, although IniA does not directly transport isoniazid from the cell (Colangeli *et al*., 2005). All these findings would be compatible with the Rv1747 transporter exporting a component of the cell wall necessary for growth of the bacillus *in vivo*. The sensitivity of the *ΔRv1747* or *ΔpknF* mutants was not however altered towards isoniazid or indeed any other of the agents tested. Neither was the transcription of the *pknF-Rv1747* operon altered by isoniazid or ethambutanol. Moreover, there were no observable changes in morphology of the cell wall as seen by electron microscopy. Thus, Rv1747 does not appear to export any component of the cell wall that is involved in formation of an observable structure.

Other significant changes in expression of cell wall-associated genes were also found in the microarray study. Thus, *ethA* and *ino1* were up-regulated in the *ΔRv1747* strain. EthA functions to activate the prodrugs ethionamide, thiacetazone and isoxyl, which all use different mechanisms to inhibit mycolic acid synthesis (Dover *et al*., 2007). Ino1 is involved in the PI biosynthetic pathway; this phospholipid is also a component of the cell envelope. The list of the top ten down-regulated genes included two genes annotated as being conserved integral membrane proteins, namely *Rv1999c* and *Rv2265*, and one gene, *Rv1382*, annotated as a probable export or membrane protein. Up-regulation of all these genes may be acting as a compensatory mechanism for the loss of the function of Rv1747.

As PknF positively regulates Rv1747 function (Spivey *et al*., 2011), it may be expected that a *ΔpknF* mutant would have a similar phenotype to a *ΔRv1747* mutant. Significantly, the *iniB* and *iniA* were also up-regulated in the *ΔpknF* mutant, correlating with the demonstration that PknF positively modulates the function of Rv1747 (Spivey *et al*., 2011). There were fewer genes whose expression level changed upon *pknF* deletion. This may be because of cross-talk between the 11 *M. tuberculosis* STPKs, which are able to cross-talk and recognise the same substrate *in vitro* (and probably also *in vivo*; Greenstein *et al*., 2005; Grundner *et al*., 2005; Molle & Kremer, 2010; Prisic *et al*., 2010). There is also a high degree of cross-reactivity between inhibitors of PknB and PknF (Lougheed *et al*., 2011).

The role of the STPK PknF that controls Rv1747 function has previously been examined in a *pknF* antisense strain (Deol *et al*., 2005). These authors described changes in cell morphology, including aberrant septum formation and reported a 16-fold increase in the uptake of d-glucose in the antisense strain. In contrast, in the present study, we did not find any morphological changes in the *ΔpknF* mutant.

Pitarque *et al*. (2008) have proposed that an unidentified transporter may be required to translocate the lipoglycans lipoarabinomannan and lipomannan, to the cell surface as the virulence of *M. tuberculosis* depends upon the export of these immunomodulatory molecules to the cell surface, as shown for the translocation of PDIMs (Sulzenbacher *et al*., 2006). The involvement in PDIM transport of an ABC transporter, DrrABC, together with the RND family protein MmpL7 (Cox *et al*., 1999; Camacho *et al*., 2001), which is a potential substrate of the STPK PknD (Pérez *et al*., 2006), is a possible paradigm for Rv1747 and PknF. Thus, the translocation of virulence-critical molecules such as lipoglycans could be a plausible explanation for the attenuation of the *ΔRv1747* mutant in mice. However, our limited analysis with two anti-lipoarabinomannan antibodies failed to find any difference in this molecule in the *ΔRv1747* mutant.

In this study, we have demonstrated that there are significant increases in gene expression in the efflux pump-related *iniBAC* genes in the Δ*Rv1747* and Δ*pknF* deletion strains. As this operon is inducible by a broad range of inhibitors of cell wall biosynthesis, we conclude that the loss of the Rv1747 transporter system affects cell wall biosynthesis and results in the accumulation of intermediates that may play a role in cell wall processes or biosynthesis, causing an induction of the *iniBAC* operon expression and implicates Rv1747 in exporting a component of the cell wall, which is required for virulence.

## Supporting Information

Fig S1 Growth inhibition assays assessing the susceptibility of WT H37Rv, Δ*pknF* and Δ*Rv1747* strains to a range of drug and stress reagents.

Fig S2 β-Galactosidase assays on the *pknF* promoterlacZ strain and pEJ414 control strain in *Mycobacterium tuberculosis* after a panel of treatments.

Fig S3 Transmission electron micrographs of *Mycobacterium tuberculosis* comparing cell wall structure in (a) WT H37Rv, Δ*Rv1747* and *Rv1747* complement strains, and (b) WT *H37Rv*, ΔpknF and *pknF* complement strains.

Fig S4 *Mycobacterium tuberculosis* whole cell ELISAs comparing the levels of ManLAM in H37Rv WT, Δ*Rv1747* and *Rv1747* complement strains.
