# Supplementary figures and images for: An attenuated mutant of the Rv1747 ATP-binding cassette transporter of Mycobacterium tuberculosis and a mutant of its cognate kinase, PknF, show increased expression of the efflux pump-related iniBAC operon
Source: FEMS Microbiol Lett. 2013 Aug 23;347(2):107–15. doi: 10.1111/1574-6968.12230 (PMC3908365; doi:10.1111/1574-6968.12230)

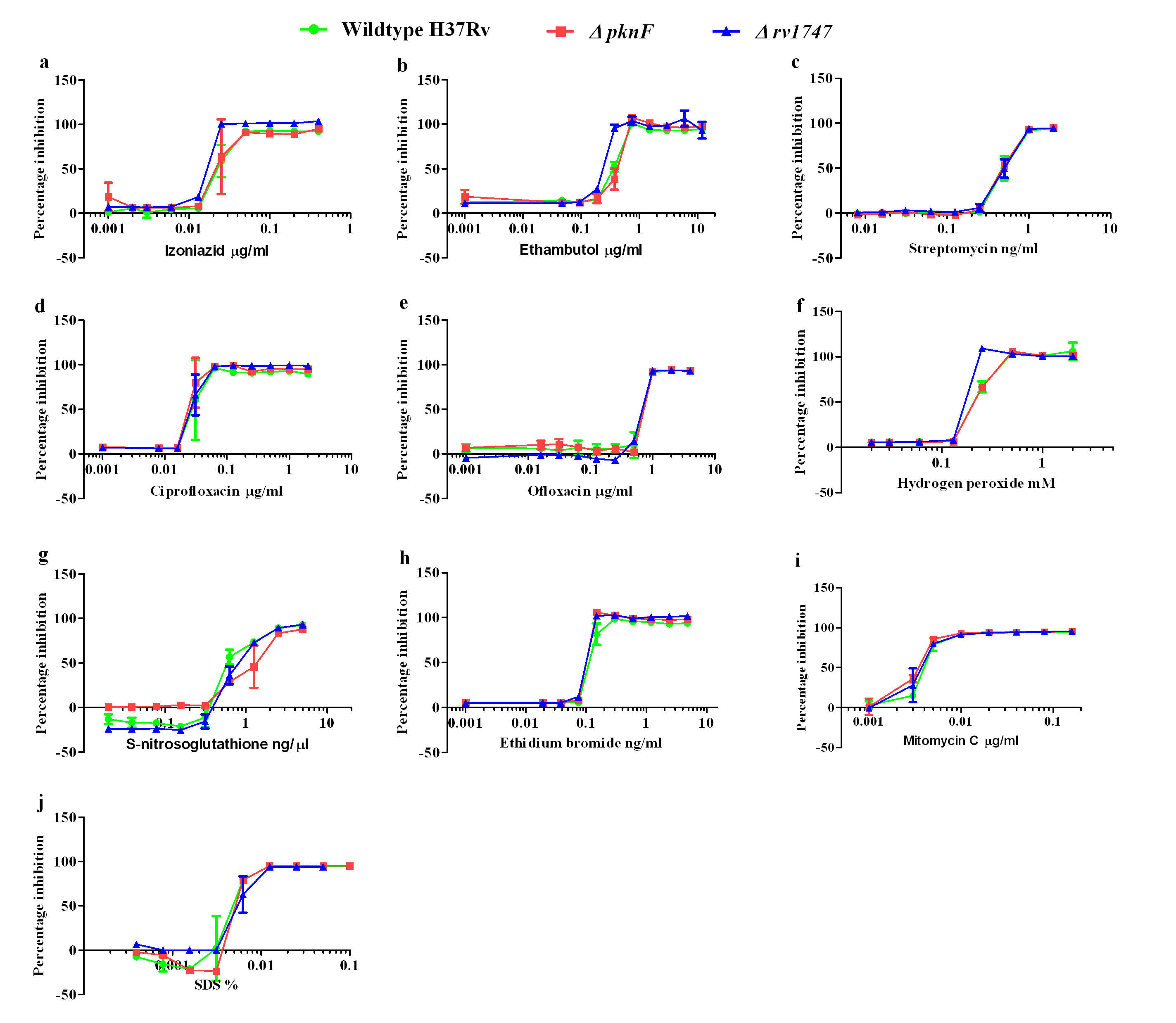

Supplement: Fig S1 — Growth inhibition assays assessing the susceptibility of WT H37Rv, ΔpknF and ΔRv1747 strains to a range of drug and stress reagents. [file fml0347-0107-sd1.tif]

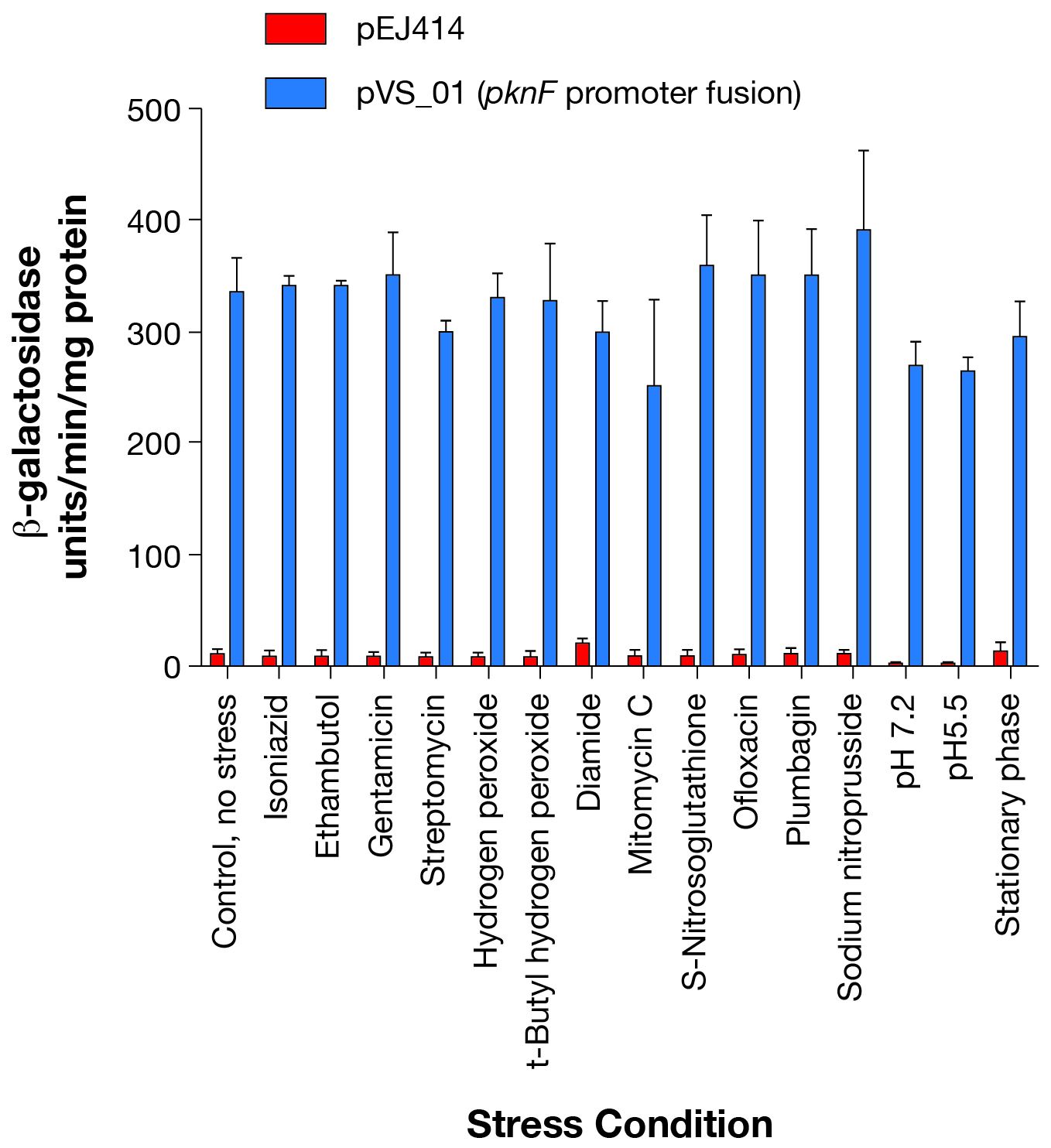

Supplement: Fig S2 — β-Galactosidase assays on the pknF promoterlacZ strain and pEJ414 control strain in Mycobacterium tuberculosis after a panel of treatments. [file fml0347-0107-sd2.tif]

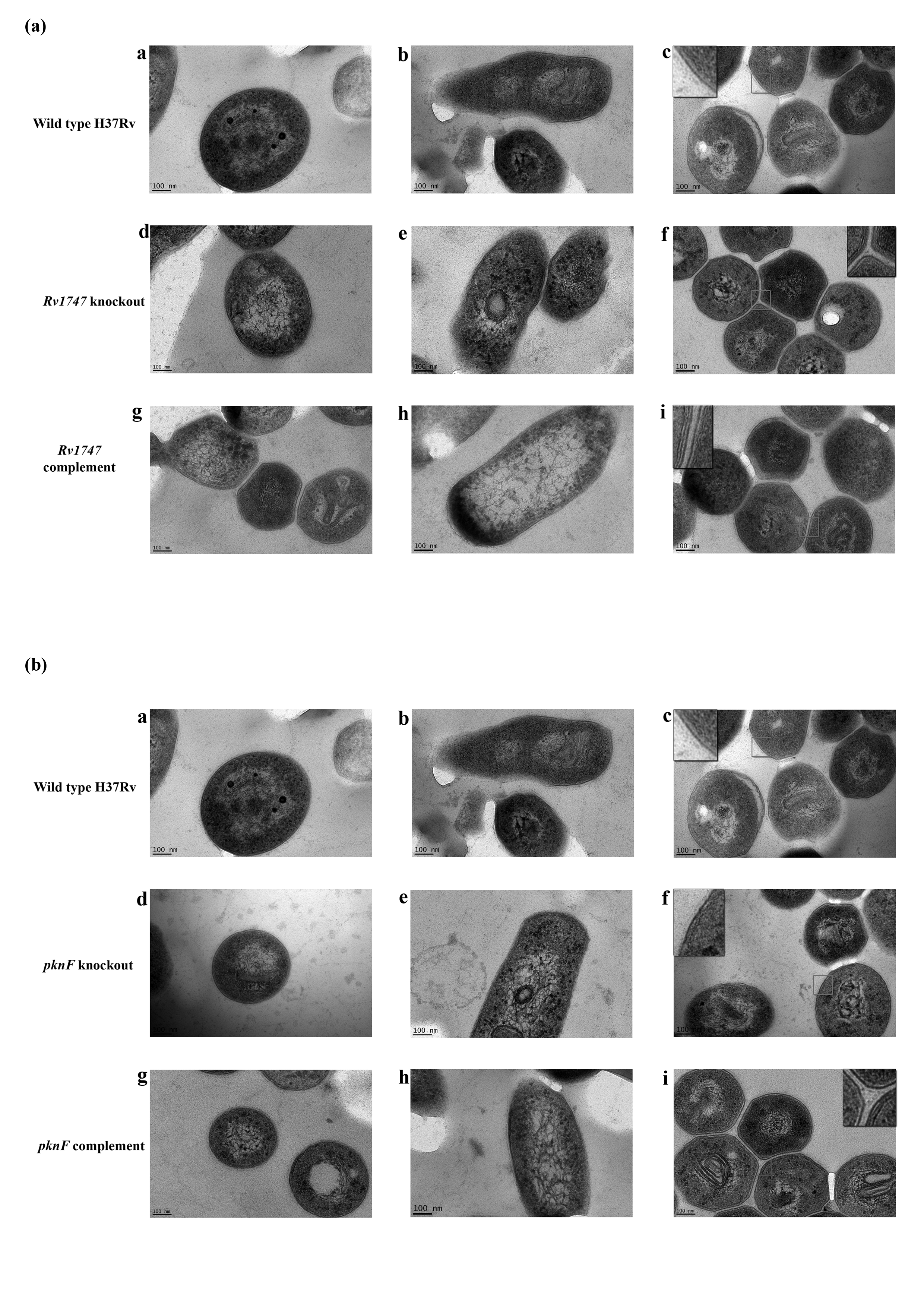

Supplement: Fig S3 — Transmission electron micrographs of Mycobacterium tuberculosis comparing cell wall structure in (a) WT H37Rv, ΔRv1747 and Rv1747 complement strains, and (b) WT H37Rv, ΔpknF and pknF complement strains. [file fml0347-0107-sd3.tif]

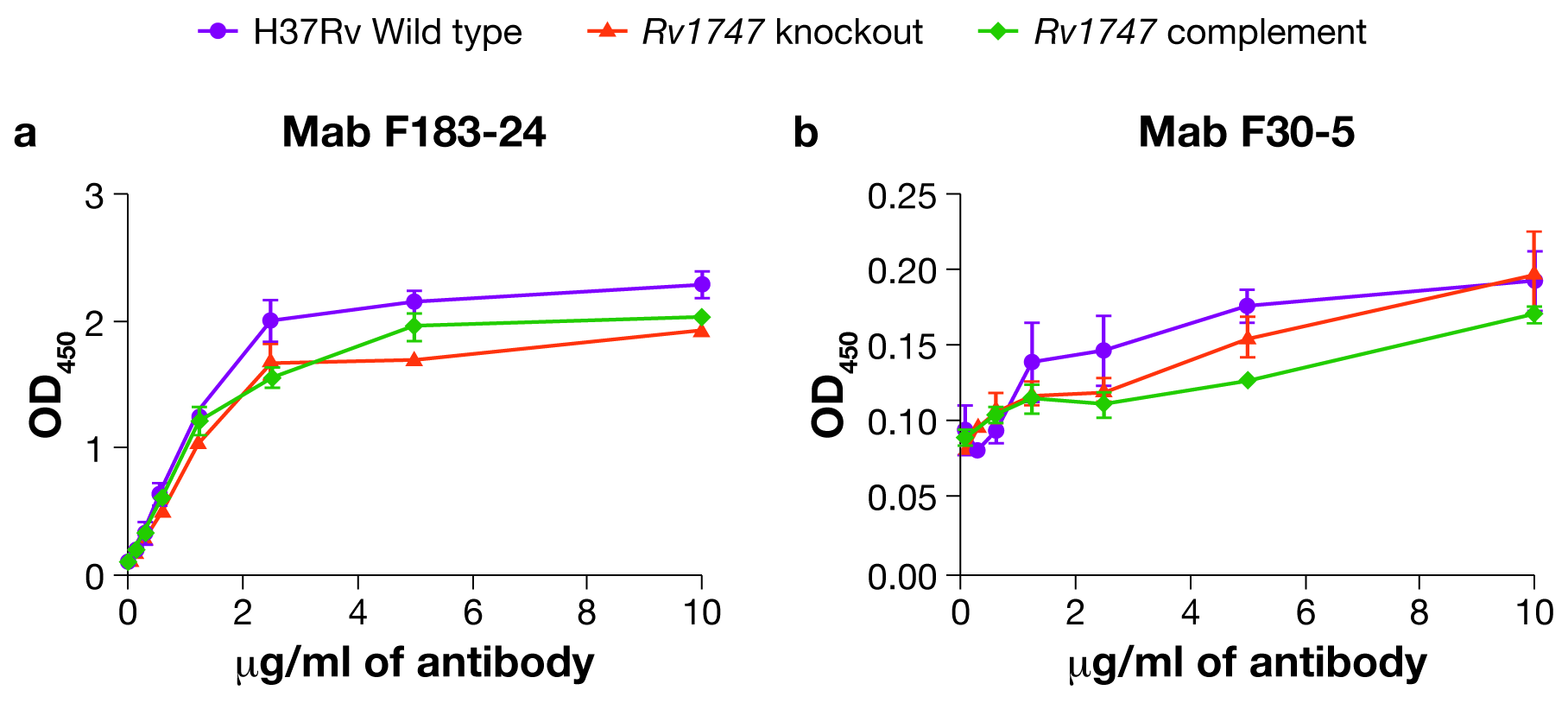

Supplement: Fig S4 — Mycobacterium tuberculosis whole cell ELISAs comparing the levels of ManLAM in H37Rv WT, ΔRv1747 and Rv1747 complement strains. [file fml0347-0107-sd4.tif]
