# Supplementary material for: An attenuated mutant of the Rv1747 ATP-binding cassette transporter of Mycobacterium tuberculosis and a mutant of its cognate kinase, PknF, show increased expression of the efflux pump-related iniBAC operon
Source: FEMS Microbiol Lett. 2013 Aug 23;347(2):107–15. doi: 10.1111/1574-6968.12230 (PMC3908365; doi:10.1111/1574-6968.12230)
Supplement: Appendix S1 — Supplementary Materials and methods [file fml0347-0107-sd5.docx]

**Supporting Information for:**

**An attenuated mutant of the Rv1747 ABC transporter of *Mycobacterium tuberculosis* and a mutant of its cognate kinase, PknF, show increased expression of the efflux-pump related *iniBAC* operon**

**Vicky L. Spivey^1*^, Rachael H. Whalan^1^, Elizabeth M.A. Hirst^2^, Stephen J. Smerdon^3^ and Roger S. Buxton^1^**

^1^Division of Mycobacterial Research, ^2^Division of Developmental Neurobiology, ^3^Division of Molecular Structure, MRC National Institute for Medical Research, Mill Hill, London NW7 1AA, United Kingdom.

**Running title:** ABC transporter mutant of *M. tuberculosis*

Corresponding author: Roger S. Buxton

Tel:(+44) 020 8816 2225, Fax: (+44) 020 8816 2225, E-mail: [rbuxton@nimr.mrc.ac.uk](mailto:rbuxton@nimr.mrc.ac.uk)

^*^Present address: Horizon Discovery Ltd., Building 7100, IQ Cambridge, Waterbeach, Cambridge, CB25 9TL, United Kingdom

**Supplementary Materials and Methods**

***M. tuberculosis* growth inhibition (Alamar Blue) assays**

This was carried out using the method of Collins and Franzblau (1997) as described previously (Lougheed *et al.*, 2009). The assay utilises the CellTiter-Blue reagent (Promega) which contains the compound resazurin. In the presence of metabolically active cells resazurin (Alamar Blue) is reduced to resorufin, a fluorescent pink compound which can be measured with an excitation wavelength of 579 nm and emission of 584 nm. Assays were carried out in 96 well plates (Greiner Bio-one). Two fold dilution series of the drug or stress reagent in 90 µl of Dubos were added to the plates. Positive (media plus bacteria) and negative (media only) control wells were included in all plates. Exponential phase *M. tuberculosis* cultures were diluted to give 1 x 10^4^ cells in 10 µl. 10 µl of this dilution was added to the appropriate wells. Plates were incubated at 37 ^o^C for five days to allow for *M. tuberculosis* growth. On day five 20 µl of CellTiter-Blue reagent was added to each well. Plates were incubated for 16 hours at 37 ^o^C to allow cells to convert the resazurin to resorufin and then the fluorescent signal was measured in a Polarstar Galaxy plate reader (BMG) as per manufacturer’s guidelines. Data were plotted as the percentage inhibition of growth compared to the positive control (media plus bacteria) versus the reagent concentration.

**Promoter reporter assay**

A promoter-*lacZ* fusion construct (pVS_01) was generated by cloning 321 bp upstream of the *pknF* transcriptional start site in front of the *lacZ* gene in pEJ414 (there are 146 bp between *pknF* and its upstream divergent gene *idi*). The plasmid is integrated into the genome of *M. tuberculosis* using pBSInt so only one copy is present per cell. This construct was sequenced and confirmed to be correct before electroporating into *M. tuberculosis*. The β-galactosidase reporter assay was used to assess the promoter activity of *pknF* under different cultural conditions. A panel of drug and stress reagents were added to exponential phase cultures (OD_600_ of 0.6 to 0.8) for two hours at 37 ^o^C with rolling. The final concentrations used were: isoniazid (0.0125 µg/ml), ethambutol (0.375 µg/ml), streptomycin (0.0625 µg/ml), gentamicin (2 µg/ml), mitomycin C (0.02 µg/ml), ofloxacin (1 µg/ml), hydrogen peroxide (2 mM), t-butyl hydrogen peroxide (0.1 mM), S-nitrosoglutathione (5 mM), diamide (10 mM), plumbagin (0.5 mM) and sodium nitroprusside (10 mM). For acid stress, cultures at an OD_600_ of between 0.25 and 0.3 were harvested and then resuspended in either Dubos at pH 7.2 or Dubos adjusted to pH 5.5 and further incubated for 24 hours. For assessing activity in stationary phase cultures were harvested when at an OD_600_ of 2.0. β-Galactosidase assays were carried out according to the method of Miller (1972). Three independent cultures were analyzed for each strain, and the mean and standard deviation of the mean were calculated.

**Electron microscopy**

*M. tuberculosis* cultures were grown to exponential phase (OD_600_ 0.6-0.8) in Dubos medium before being resuspended in 2% glutaraldehyde / 2% paraformaldehyde in 0.1 M sodium cacodylate buffer (pH 7.2) for 18 hours in order to fix the cells. Cells were then prepared and then stained with aqueous uranyl acetate and Reynold's lead citrate for transmission electron microscopy. Images were then taken with a Gatan Orius 1000 CCD camera at x 25,000 magnification. Ten images were taken per section and three sections were examined per strain.

**Lipoarabinomannan (LAM) analysis**

Whole cell ELISAs were performed using antibodies raised against ManLAM, Mab F183-24 and Mab F30-5 (gifts from Ben Appelmelk and Jeroen Geurtsen). *M. tuberculosis* cell suspensions were adsorbed to 96-well ELISA plates, incubated with the primary antibodies and an IgM secondary antibody peroxidise conjugate added to each well. After addition of 3,3’,5,5’-tetramethylbenzidine (TMB), colour formation was monitored and the reaction stopped by adding H_2_SO_4_ producing a yellow colour. Absorbance was measured at 450 nm.

References

Collins L & Franzblau SG (1997) Microplate alamar blue assay versus BACTEC 460 system for high-throughput screening of compounds against *Mycobacterium tuberculosis* and *Mycobacterium avium*. *Antimicrob Agents Chemother* **41**: 1004-1009.

Lougheed KEA, Taylor DL, Osborne SA, Bryans JS & Buxton RS (2009) New anti-tuberculosis agents amongst known drugs. *Tuberculosis* **89**: 364-370.

Miller JH (1972) *Experiments in Molecular Genetics*. Cold Spring Harbor Press, New York.
